# Supplementary material for: An examination of difficulties accessing surgical care in Canada from 2005-2014: Results from the Canadian Community Health Survey
Source: PLoS One. 2020 Oct 21;15(10):e0240083. doi: 10.1371/journal.pone.0240083 (PMC7577481; doi:10.1371/journal.pone.0240083)
Supplement: S1 Table — (DOC) [file pone.0240083.s001.doc]

| Supplemental Table 1  *Provinces that completed the ACC and WTM modules according to survey cycle* | | |
| --- | --- | --- |
| Year | ACC module | WTM module |
| 2005/2006 | New Brunswick | New Brunswick |
| 2007 | Ontario | Ontario |
| 2008 | New Brunswick | -- |
| 2009 | Ontario | Ontario |
| 2010 | Ontario | Ontario |
| 2011 | British Colombia | -- |
| 2012 | Newfoundland, New Brunswick, British Colombia | Newfoundland |
| 2013 | Nova Scotia | -- |
| 2014 | Newfoundland, Nova Scotia, New Brunswick | Newfoundland |
| *Note.* Provinces had the opportunity to opt out of answering certain modules of the Canadian Community Health Survey; ACC = Access to Health Care Services; WTM= Waiting Times | | |
