# Supplementary material for: An examination of difficulties accessing surgical care in Canada from 2005-2014: Results from the Canadian Community Health Survey
Source: PLoS One. 2020 Oct 21;15(10):e0240083. doi: 10.1371/journal.pone.0240083 (PMC7577481; doi:10.1371/journal.pone.0240083)
Supplement: S1 Appendix — (DOC) [file pone.0240083.s002.doc]

Appendix A

Response Rates

| CCHS annual component response rates from 2005-2014 | |
| --- | --- |
| Year | Response rate |
| 2005-2006 | 78.9% |
| 2007 | 77.6% |
| 2008 | 75.2% |
| 2009 | 73.2% |
| 2010 | 71.5% |
| 2011 | 69.8% |
| 2012 | 67.0% |
| 2013 | 66.8% |
| 2014 | 65.6% |
