# Supplementary material for: An examination of difficulties accessing surgical care in Canada from 2005-2014: Results from the Canadian Community Health Survey
Source: PLoS One. 2020 Oct 21;15(10):e0240083. doi: 10.1371/journal.pone.0240083 (PMC7577481; doi:10.1371/journal.pone.0240083)
Supplement: S2 Appendix — (DOC) [file pone.0240083.s003.doc]

Appendix B

Access to Health Care Services (ACC) and Waiting Times (WTM) Questions

ACC Module

The following questions are about any surgery not provided in an emergency that you may have required, such as cardiac surgery, joint surgery, caesarean sections and cataract surgery, excluding laser eye surgery.

1. In the past 12 months, did you require any non-emergency surgery?
2. In the past 12 months, did you ever experience any difficulties getting the surgery you needed?
3. What types of difficulties did you experience?

WTM Module

Now some additional questions about your experiences waiting for health care services. You mentioned that in the past 12 months you required non-emergency surgery.

1. What type of surgery did you require? If you had more than one in the past 12 months, please answer for the most recent surgery.
2. Was your surgery cancelled or postponed at any time? Was it cancelled or postponed by: yourself?...the surgeon?...the hospital?
3. Did the surgery require an overnight hospital stay?
4. How long did you have to wait between when you and the surgeon decided to go ahead with surgery and the day of surgery?
5. In your view, was the waiting time acceptable or not acceptable?
6. In this particular case, what do you think is an acceptable waiting time?
